# Serrated Pathway Adenocarcinomas: Molecular and Immunohistochemical Insights into Their Recognition

**DOI:** 10.1371/journal.pone.0057699

**Published:** 2013-03-04

**Authors:** Simona Gurzu, Zoltan Szentirmay, Erika Toth, Tivadar Bara, Tivadar Bara, Ioan Jung

**Affiliations:** 1 Department of Pathology, University of Medicine and Pharmacy of Targu-Mures, Targu-Mures, Romania; 2 Department of Pathology, National Institute of Oncology, Budapest, Hungary; 3 Department of Surgery, University of Medicine and Pharmacy of Targu-Mures, Targu-Mures, Romania; Sapporo Medical University, Japan

## Abstract

**Introduction:**

Colorectal adenocarcinomas (CRC) developed through serrated pathway seem to present particular behavior compared with the non-serrated ones, but recognition of them is difficult to do. The aim of our paper was to establish some criteria to facilitate their identification.

**Materials and Methods:**

In 170 consecutive CRCs, we performed immunohistochemical staining with Cytokeratin 7 (CK7) and Cytokeratin 20 (CK20) and also with p53 and MLH-1. At the same time, we analyzed BRAF and K-ras mutations and the microsatellite status of CRC.

**Results:**

26.47% of cases expressed CK7, and 16.47% were CK20-negative. Diffuse positivity for CK7 was associated in the proximal colon with CK20 negativity or weak positivity, BRAF mutations, lack of K-ras mutations, and p53 and MLH-1 negativity. All these cases were microsatellite-unstable and were diagnosed in stage II. Those cases from the distal colon and rectum that expressed CK7 were K-ras-mutated and had low p53 index and MLH-1 positivity, independent of the CK20 expression.

**Conclusions:**

CK7, associated with MLH-1 and p53 expression, and also with the microsatellite status, BRAF and K-ras pattern, might be used to identify the CRC potentially going through serrated pathway. The serrated pathway adenocarcinomas of the proximal colon that do not display the morphological features of this pattern are more frequent CK7+/p53−/MLH-1−/BRAF-mutated/K-ras-wt/MSI cases, but those located in the distal colorectal segments seem to be CK7+/CK20+/p53−/MLH-1+/BRAF wt/K-ras-mutated/MSS cases.

## Introduction

Serrated adenomas were first described in 1990 as colorectal polyps with mixed histologic features of hyperplastic and adenomatous component [1], [2]. Their malignant transformation can lead to serrated adenocarcinomas, which seem to have a distinct behavior and probably respond to a specific therapy [3].

Two types of serrated adenomas are described: the traditional polypoid type, which is usually smaller and located in the distal colon [4], and the non-polypoid, or sessile serrated adenomas, which can occur throughout the colon, but the proximal ones are larger than their counterparts [2], [5].

The serrated neoplasia pathway still remains a non-elucidated and controversial subject. Some authors admit that mutations of p53 and APC (Adenomatous Polyposis Coli) genes are not specific for these tumors but microsatellite instability is observed more frequently than in non-serrated adenocarcinomas [2], [3], [6]. At the same time, specific pathway seems to be involved in different colon segments. In right-sided sessile serrated adenomas, MSI (microsatelite instability) pattern, immunohistochemical loss of MLH1 expression and BRAF mutations are frequently observed, but K-ras mutations occur in fewer than 10% of the cases [7]. Most of the traditional polypoid serrated adenomas from distal colon are MSS (microsatellite stable), 80% of them are associated with MLH1 methylation and K-ras mutations [6], but BRAF mutations are less frequent [6], [7].

Based on these observations, Jass revealed that 20% of CRCs are sporadic serrated adenocarcinomas: 12% occur from the right-sided sessile serrated adenomas and 8% occur from the polypoid distal-located serrated adenomas [8]. The correct identification of these types remains a challenging subject with possible prognostic and therapeutic impact.

The main objectives of our paper were to analyze the clinicopathological, immunohistochemical, and molecular characteristics of CRC, based on the previously described features of serrated adenocarcinomas, and to identify the molecular and immunohistochemical criteria that can be used to recognize the possible serrated pathway. In one of our previous studies, based on an investigation of 52 cases, we proved that Cytokeratin 7 (CK7) was positive in the MSI right-sided BRAF-mutated colon carcinomas, which, probably, were serrated adenocarcinomas [3]. No other studies were published regarding the correlation between MSI, BRAF mutation, and CK7/CK20 immunophenotype. In this paper, we increased the number of cases to 170 and also analyzed the K-ras status to determine the practical importance of the correlation between immunohistochemical and molecular profiles of CRC.

## Materials and Methods

### Patients and Tissue Samples

Our study included 170 consecutive cases of colorectal adenocarcinomas (87 from proximal and 83 from distal colon and rectum), 10 hyperplastic polyps, 10 sessile serrated adenomas, and 10 traditional serrated adenomas. Processing of the cases was approved by the Ethical Committee of University of Medicine and Pharmacy of Targu-Mures, Romania. Written informed consent was obtained from each patient prior to beginning any research.

### Immunohistochemical Staining and Scoring

In case of colorectal adenocarcinomas, immunohistochemical staining was performed with Cytokeratin 7 (clone OV-TL 12/30, D-Line, LabVision), Cytokeratin 20 (clone Q6, D-Line, LabVision), p53 (clone DO-7, D-Line, LabVision) and MLH-1 (monoclonal, BD Biosciences). UltraVision system by D-Line, LabVision (Fremont, CA, USA), was used in analyzing immunohistochemical reactions in formalin-fixed, paraffin-embedded tissues. The 5-µm-thick sections were deparaffinized, rehydrated, and incubated at 100°C with pepsin (Cytokeratins, MLH-1) and citrate (p53). The incubation with primary antibodies was realized for 60 minutes. DAB (Diaminobenzidine, Dako) was used as chromogen. All slides were counterstained with Mayer’s Hematoxylin. In case of hyperplastic polyps, sessile serrated adenomas, and traditional serrated adenomas, Cytokeratin 7 and Cytokeratin 20 were also analyzed.

For negative controls, incubation was performed with omission of the specific antibodies. To prevent non-specific staining, endogenous biotin and enzyme interference we used for each cases negative and positive controls and also an external quality control was performed. The pre-immunohistochemistry steps were also controled, including the formalin neutralization and fixation and paraffin-embedded processes. The non-specific negative reagent control in place of primary antibody was used to evaluate the non-specific staining, according to the manufacturer’s instructions.

CK7 and CK20 expressions were evaluated in the cell membrane and cytoplasm, and p53 ones was analyzed in the nuclei of the tumor cells. We considered those positive cases that expressed CK7 and CK20 in more than 5% of the cells. Focal positivity was considered as the presence of antibody expression in 5–50% of the tumor cells; the other cases were considered to have diffuse positivity, as previously described [9]. Those cases that expressed p53 in more than 10% of the nuclei were considered p53-positive; the same criteria were used for MLH-1.

### Molecular Analysis

For the molecular examinations, DNA was extracted from microdissected formalin-fixed, paraffin-embedded tissues. Real-Time PCR-based DNA (Roche GmbH, Mannheim, Germany) was used. DNA isolation was done using the High Pure PCR Template Preparation Kit (Roche GmbH, Mannheim, Germany). DNA quality was checked by electrophoresis. DNA concentration was determined using the Nanodrop machine. In each case the specifically dilutions were performed to have a 50–100 ng template DNA.

To determine microsatellite instability, we used the mononucleotide markers BAT25 and BAT26 (Roche) and the method of High-Resolution Melting analysis. Specifically primers, previously published [10], were used for BAT25 (forward 5′ - TCGCCTCCAAGAATGTAAGT - 3′ and reverse 5′ - TCTGCATTTTAACTATGGCTC - 3′) and BAT26 (forward 5′ - TGACTACTTTTGACTTCAGCC - 3′ and reverse 5′ - AACCATTCAACATTTTTAACCC-3′). For BAT26 the average melting peak value was 51.2°C for MSS respectively 45.5°C for MSI. For BAT25 the average values were 45.1°C for MSS respectively 42.4°C for MSI. Cases that showed instability with both nucleotides were considered to be MSI-H (high frequency microsatellite instability), the others being MSS (microsatellite stable). No cases with instability in one of the two markers have been identified.

DNA sequencing method was used to test the BRAF and K-ras mutations. Substitution of glutamic acid for valine in codon 600 was considered to be BRAF V600E mutation. For K-ras, both codon 12 and codon 13 mutations were analyzed.

### Statistical Analysis

The results were evaluated using the Graph Pad statistical software. *p* values less than 0.05 with 95% confidence interval were considered significant. We take into consideration the following covariates regarding the patients’ characteristics and their molecular and immunohistochemical features: age, gender, tumor location, histologic type and grade, tumor markers (CK20, CK7, p53, MLH-1), and the molecular status (MSI/MSS, BRAF and k-RAS mutations). To present the patients’ characteristics we used the descriptive statistic. The patients’ age was expressed in mean ± standard deviation and was analyzed with Pearson’s or student’s t-test. For multiple associations the Fisher exact test was used, and the correlations between two variables were performed using the Mann-Whitney test.

Multivariate analysis involved the binary logistic regression model. Each immunohistochemical and molecular variables that achieved statistical significance in the univariate analysis were subsequently included in the multivariate analysis to assess the independent correlation between a categorical variable and CK7 positivity. The final purpose was evaluate the probability that a CRC with a particular molecular status, in relationship to the CK7 expression, will have a possible serrated pathway.

## Results

### Clinico-pathological Data

The median age of the patients included in this study was 59.58±11.57 years, ranging between 23 and 82 years old. From the 170 cases, 120 (70.59%) were males and 50 (29.41%) were females.

Most of the cases (56.78%) were diagnosed in stage III, with lymph node metastases; the other cases were diagnosed in stage II (22.14%), non-metastatic cases, and stage IV (21.08%).

In this study, 25.30% cases were mucinous adenocarcinomas, while others were non-mucinous type, further characterized as well (11.76%), moderately (47.06%), or poorly differentiated (15.88%).

Synchronous residual serrated adenoma either specifical histological criteria of serrated adenocarcinomas, such as epithelial serrations were not identified in these cases. Most of the MSI-H cases were poorly differentiated or presented mucinous component, they did not showed areas with necrosis, eosinophilic cytoplasm and vesicular nuclei were inconstantly present but these criteria were not enough to consider these cases serrated type.

Expression of cytokeratins and the molecular status of colorectal carcinomas.

### Descriptive Statistics

From the 170 cases, 14 (8.23%) had BRAF mutations - 13 being located in the proximal colon and one in the rectum. Microsatellite instability (MSI-H) was detected in 16 cases (9.41%) - all being right-sided poorly differentiated or mucinous adenocarcinomas.

From the 71 cases with K-ras codon 12 mutations (41.76% of CRCs), 47 were located in the distal colon and rectum, and 24 were in the proximal colon. From the 170 analyzed CRC, 10 presented K-ras codon 13 mutations (5.88% of CRCs), seven being located in the distal colon and rectum, and three in the proximal colon (Table 1).

**Table 1:** The correlation between clinico-pathological parameters, CK7/CK20 immunoprofile and molecular status of colorectal carcinomas.

Variable (n = 170)	Cytokeratin 7 expression			
	negative (n = 125)	focal positive (n = 30)	diffuse positive (n = 15)	P
**Age** (mean±SD, years)	60.13±10.86	57.14±14.47	56.4±12.51	0.42
**Male:female ratio**	3∶1	2∶1	1∶0	0.80
**Localization**				
proximal (n = 54)	36%	26%	56%	***0.02***
distal/rectum (n = 116)	64%	74%	44%	
**Histologic type/grade**				
G1 (n = 20)	14%	18%	13%	
G2 (n = 80)	51%	68%	13%	***<0.0001***
G3 (n = 27)	19%	5%	48%	
Mucinous cc. (n = 43)	16%	9%	26%	
**CK20 expression**				
Negative (n = 28)	15%	5%	50%	
Focal positive (n = 86)	52%	72%	29%	***<0.0001***
Diffuse positive (n = 56)	33%	23%	21%	
**p53 expression**				
negative (n = 80)	48%	43%	44%	
<50% (n = 26)	14%	19%	22%	0.66
>50% (n = 64)	38%	38%	34%	
**MLH-1 expression**				
Negative (n = 22)	27%	19%	70%	***<0.0001***
Positive (n = 148)	73%	81%	30%	
**Microsatellite status**				
MSI (n = 16)	15%	5%	80%	***0.006***
MSS (n = 154)	85%	95%	20%	
**BRAF status**				
wt (n = 156)	79%	95%	50%	***<0.0001***
MUT (n = 14)	21%	5%	50%	
**K-ras codon 12 status**				
wt (n = 99)	63%	57%	44%	***0.02***
MUT (n = 71)	37%	43%	56%	
**K-ras codon 13 status**				
wt (n = 160)	61%	49%	40%	***0.01***
MUT (n = 10)	39%	51%	60%	

(G = tumor grade; cc = carcinoma; MSI = Microsatellite Instability; MSS = Microsatelite Stable; wt = wild type; MUT = mutant;).

Although CK7−/CK20+ is the immunophenotype quite specific for CRC, in our study, 26.47% of cases were CK7-positive, and 16.47% did not express CK20.

### Univariate Analysis

**Figure 1:**
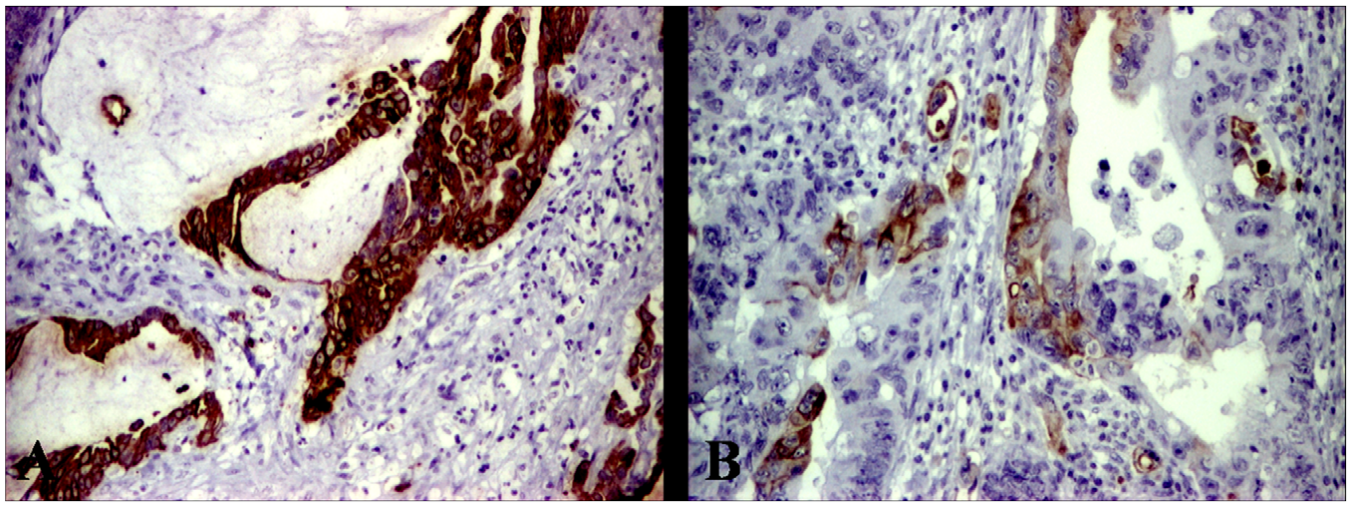
Immunoexpression of Cytokeratin 7 in case of possible serrated pathway colorectal adenocarcinomas. Most of mucinous carcinomas present diffuse positivity (A) but the moderately-differentiated ones show focal positivity (B).

The CRCs with diffuse positivity for CK7 were more frequently right-sided poorly differentiated or mucinous adenocarcinomas (Figure 1), and 50% of them associated CK20 negativity (Table 1). Those located in the proximal colon were MSI cases with BRAF mutations, but the CK7-positive cases from distal colon and rectum were K-ras-mutated cases with low p53 index and MLH-1 positivity (Table 2).

**Table 2:** The differences between proximally and distally located colorectal carcinomas with K-ras codon 12 mutations.

Variable (n = 71)	Localization of K-ras mutated carcinomas		
	Proximal colon (n = 24)	Distal colon and rectum (n = 47)	p
**Age** (mean±SD, years)	60.23±9.35	54.41±11.46	0.11
**Male:female ratio**	6∶1	4∶1	1.00
**Histologic type/grade**			
G1 (n = 17)	31%	21%	
G2 (n = 39)	46%	59%	0.19
G3 (n = 7)	8%	10%	
Mucinous cc. (n = 8)	15%	10%	
**CK20 expression**			
Negative (n = 30)	17%	12%	0.31
Positive (n = 41)	83%	88%	
**CK7 expression**			
Negative (n = 49)	85%	40%	***<0.0001***
Positive (n = 22)	15%	60%	
**p53 expression**			
Negative (n = 42)	85%	36%	***<0.0001***
Positive (n = 29)	15%	64%	
**MLH-1 expression**			
Negative (n = 9)	23%	7%	***0.001***
Positive (n = 62)	77%	93%	
**Microsatellite status**			
MSI-H (n = 1)	8%	0%	NA
MSS (n = 70)	92%	100%	

(G = tumor grade; cc = carcinoma; MSI = Microsatellite Instability; MSS = Microsatelite Stable; wt = wild type; MUT = mutant).

All 13 right-sided BRAF-mutated carcinomas were MSI, poorly differentiated, or mucinous type; p53- and MLH-1-negative; and without K-ras mutations. All of them were diagnosed in stage II and presented CK7 positivity. At the same time, 8 from the 13 cases did not express CK20.

All hyperplastic polyps and sessile serrated adenomas presented the immunophenotype CK7+/CK20+. The ten traditional serrated adenomas expressed CK20 but CK7 was negative in all of the examined cases.

### Multivariate Analysis

For the carcinomas located in the proximal colon, the multivariate analysis indicated that MLH1 negativity (OR = 0.39, 95%CI, p = 0.034), BRAF mutations (OR = 0.37, 95%CI, p = 0.0045), absence of K-ras mutation (OR = 0.34, 95%CI, p = 0.0012) and MSI (OR = 1.24, 95%CI, p = 0.019) presented an independent correlation with the CK7 positivity. In case of carcinomas located in the distal colorectal segments, the factors that achieved independent statistical significance were MLH1 positivity (OR = 1.95, 95%CI, p = 0.042), absence of BRAF mutation (OR = 0.32, 95%CI, p = 0.0037), K-ras mutation (OR = 1.67, 95%CI, p = 0.025) and MSS (OR = 0.37, 95%CI, p = 0.0046). Although p53 and CK20 expressions presented statistical significance in univariate analysis, none of them proved to be independent factors in the multivariate analysis (OR = 1.39, 95%CI, p = 0.067 respectively OR = 2.78, 95%CI, p = 0.078).

## Discussion

Some of the most recent researchers showed that more than 15% of CRCs express CK7 and/or are CK20-negative [11], [12]. This aberrant immunophenotype seems to be related to microsatellite instability [13], being more frequent in BRAF-mutated MSI cases localized in the proximal colon [3].

In this study, we tried to examine and to emphasize the differences between proximal and distal colon segments regarding the possible serrated neoplasia patway, based on the clinico-pathological, immunohistochemical and molecular profile of these cases, and also on the literature data. One hand, all MSI cases that were located in the proximal colon, p53/MLH-1-negative, and BRAF-mutated/K-ras wt (wild type) were diffusely positive for CK7 and presented either focal positivity or negativity for CK20. These associated features are characteristics of a serrated neoplasia pathway of the proximal colon [14], [15] and could facilitate identification of those serrated carcinomas which do not associate synchronous serrated adenomas, as in our lot of patients. Our observations are based on some studies such the Tatsumi *et al.* ones; they reported a diffuse CK7 positivity in colorectal adenocarcinomas developing in serrated adenoma [16]. At the same time, the V600E BRAF muation is considered to be exclusively associated with sporadic MSI-CRC, which are usually K-ras wt [17]. The results of the present research, correlated with the previous ones, sustain the idea that serrated neoplasia pathway of the proximal colon could be characteristic for the BRAF-mutated/K-ras wt/p53 negative/MLH-1 negative/CK7 positive cases, independently by the CK20 expression.

On the other hand, the CK7-positive cases of the left colon were K-ras-mutated/BRAF-wt, and p53-negative/MLH-1-positive, and most of them also expressed CK20. According to Jass et al., these features are characteristics of traditional polypoid serrated adenocarcinomas located in the distal colon [6] and have an aggressive course [18].

Our study proved that most of right-sided colonic adenocarcinomas potentially going through the serrated pathway, are CK7+/p53−/MLH-1−/BRAF-mutated/K-ras-wt/MSI, but those located in the distal colorectal segments are more frequently CK7+/CK20+/p53−/MLH-1+/BRAF wt/K-ras-mutated/MSS cases.

These characteristics could also be very useful in the daily practice to select the right-sided cases with high probability for MSI-BRAF mutated status which seem to be sporadic MSI cases with better prognosis and low risk of distant metastases than MSI-BRAF wt or MSS-BRAF mut ones [17], [19]. At the same time, the MSI-BRAF mutated cases with loss of MLH-1 immunoexpression seem to respond to oxaliplatin-based therapy [17]. In the distal colon and rectum, CK7 positivity could also help to select the cases with high probability for K-ras mutation that do not respond at anti-EGFR drugs.
